# CleanCam: A labelled underwater image dataset for camera-viewport fouling assessment in aquaculture monitoring

**DOI:** 10.1016/j.dib.2026.113137

**Published:** 2026-08-10

**Authors:** Khoa Nguyen, Anh Hoang, Nhat-Anh Tran, Nguyet-Anh Tran, Minh-Hoang Pham, Khoi Phan, Van-Dinh Nguyen, Dzung V. Dinh, Cuong Do

**Affiliations:** a College of Engineering and Computer Science, VinUniversity, Vinhomes Ocean Park, Gia Lam District, Hanoi, 100000, Vietnam; b Smart Green Transformation Center (GREEN-X), VinUniversity, Vinhomes Ocean Park, Gia Lam District, Hanoi, 100000, Vietnam

**Keywords:** Aquaculture, Underwater imaging, Biofouling, Camera viewport, Ordinal labels, Synthetic augmentation, Reproducibility, Image quality control

## Abstract

CleanCam is a labelled underwater image dataset for assessing fouling on camera viewports in aquaculture monitoring. Real images were sampled every 10 s from fixed underwater videos recorded by two camera nodes in golden pompano (*Trachinotus blochii*) rearing tanks at the Research Institute for Aquaculture No. 1 in Hai Phong, Viet Nam. The real subset contains 18,972 RGB JPEG images collected across 20 effective collection days and annotated on a five-level ordinal scale from L1 clean to L5 heavy obstruction. The labels describe material attached to the camera viewport, rather than generic image quality. Temporal context was used during annotation to distinguish persistent viewport deposits from turbidity, suspended particles, lighting variation, and other water-column effects. The release also includes 3,600 split-consistent synthetic images for Levels 3 to 5, giving 22,572 images in total. The Zenodo package provides image folders, image-level metadata, synthetic provenance records, deterministic capture-disjoint train, validation, and test splits, a data dictionary, release manifests, and documentation. The accompanying public code reproduces the manuscript figures and tables and supports release validation, dataset characterization, synthetic-subset analysis, and reference classification workflows. CleanCam can be reused for ordinal image classification, cleaning/no-cleaning threshold studies, synthetic augmentation experiments, leakage-aware benchmarking, and image-stream quality control in long-term aquatic monitoring systems.

## Graphical abstract

**Figure:**
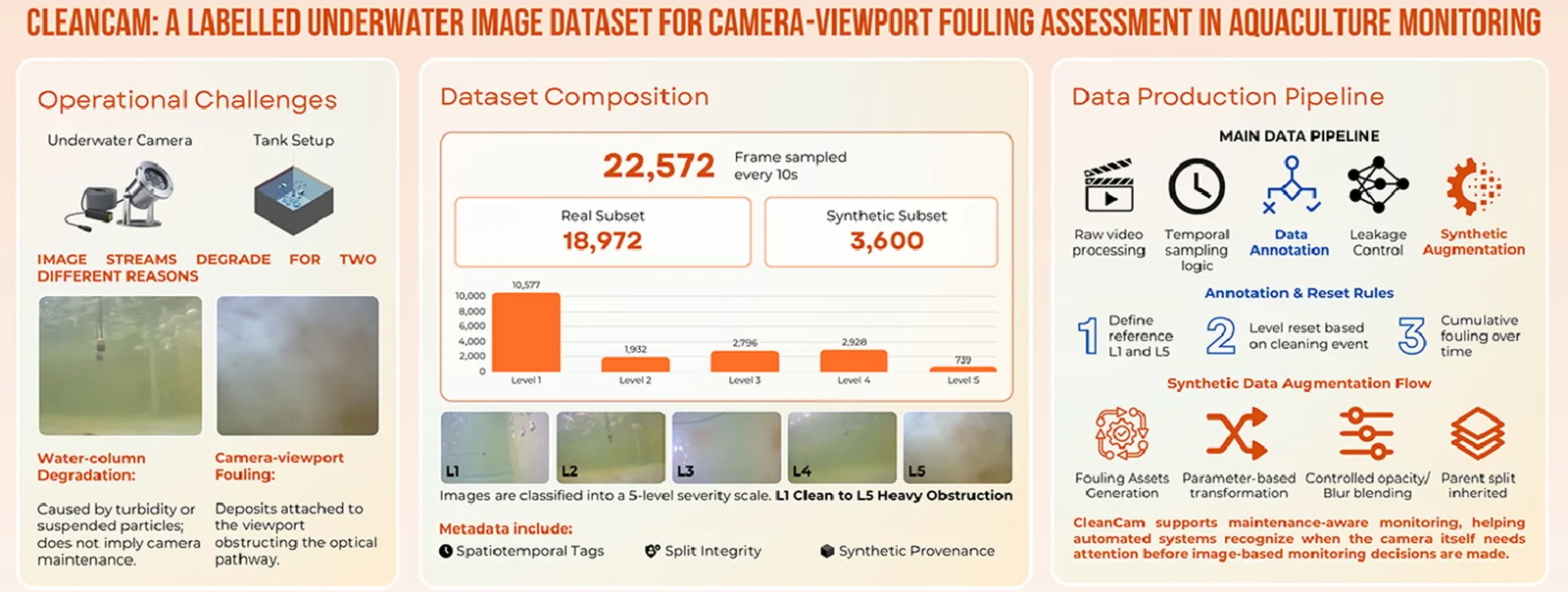

Specifications Table

**Table:** 

Subject	Computer Vision and Pattern Recognition
Specific subject area	Underwater image data for camera-viewport fouling assessment in aquaculture monitoring
Type of data	Image; Table; Figure; Code JPEG images; CSV metadata and split files; JSON build summary; Python scripts; Markdown documentation Raw; filtered; processed; analyzed
Data collection	Real images were sampled every 10 s from approximately 53 h of 3072 × 2048 underwater videos recorded by two fixed Barlus IPC5MPIR-BX10 cameras mounted at approximately 1 m depth in golden pompano rearing tanks from 30 August to 19 September 2025. Frames were curated, annotated on a five-level viewport-fouling scale using temporal context, and split by capture. Synthetic images for Levels 3-5 were generated from real parent images with recorded deposit-overlay parameters.
Data source location	Institution: Research Institute for Aquaculture No. 1 City/Town/Region: Hai Phong Country: Viet Nam
Data accessibility	Repository name: Zenodo Data identification number: 10.5281/zenodo.21515620 [1] Direct URL to data: https://doi.org/10.5281/zenodo.21515620 Instructions for accessing these data: Download CleanCam_v2.zip, extract the archive, and use the metadata and official split files in CleanCam_v2/. No access request or Data Usage Agreement is required. Image paths are relative to the extracted release root. License: Creative Commons Attribution 4.0 International (CC BY 4.0).
Code accessibility	Public code repository: GitHub, https://github.com/khoa288/CleanCam [2] Instructions for accessing the code: Clone or download the repository. The pinned core environment, quickstart, release validator, data dictionary, fixed example identifiers, and one-command manuscript reproduction script are included.
Related research article	None.

## 1 Value of the Data

• The data provide field-captured underwater examples of camera-viewport surface deposits under aquaculture monitoring conditions, with labels tied to cleaning severity rather than to generic image quality.
• The five-level ordinal labels can be reused to train and evaluate models that distinguish persistent viewport deposits from turbidity, suspended particles, haze, lighting variation, and other water-column effects.
• The metadata support grouped analysis by camera, date, session, elapsed time, capture identifier, label, split, image path, and synthetic parentage.
• The official capture-disjoint train, validation, and test files provide a common protocol for ordinal classification, cleaning/no-cleaning thresholds, and image-stream quality-control tasks.
• The synthetic subset and generator records allow controlled augmentation experiments for moderate-to-heavy viewport obstruction while preserving the link between each synthetic image and its real parent image. The data can also support camera-health studies in underwater robotics, aquatic environmental monitoring, and computer vision under degraded imaging conditions.

## 2 Background

Underwater cameras are increasingly used in aquaculture to observe fish, equipment, feeding, welfare proxies, and routine operational conditions [[3], [4], [5]]. In long-term deployments, image degradation can arise from the water column, including turbidity, suspended particles, low illumination, and backscatter, or from material attached to the camera viewport. These sources of degradation can look similar in isolated frames but imply different operational responses. Water-column degradation may require delayed interpretation or water-quality checks, whereas viewport deposits indicate a camera-maintenance problem.

Biofouling and attached deposits are recognized operational burdens in marine aquaculture and underwater sensing [[6], [7], [8], [9]]. Optical instruments and underwater cameras are particularly sensitive because the measurement path depends on a transparent surface remaining clear [10,11]. CleanCam was compiled to document this specific maintenance state using fixed underwater cameras in golden pompano rearing tanks.

Existing underwater-image datasets and benchmarks usually target general underwater visibility, color attenuation, haze, blur, low contrast, image enhancement, or synthetic water-column degradation [[12], [13], [14], [15], [16]]. CleanCam instead labels the visible condition of the camera viewport. The release therefore complements existing underwater-image resources by providing camera-condition labels, capture-disjoint splits, synthetic provenance, and reproducibility code for cleaning-decision modelling and image-stream quality control.

## 3 Data Description

The data release is distributed as CleanCam_v2.zip on Zenodo [1]. After extraction, the top-level folder is CleanCam_v2/. The package contains image folders, metadata tables, official split files, synthetic-generation records, release and validation scripts, release manifests, a data dictionary, and documentation. The public code repository provides the scripts used to reproduce the manuscript figures and tables [2].

The real subset covers 20 effective collection days and two camera nodes with morning and afternoon sessions (Fig. 3). L1 accounts for 55.8% of the real images; L2, L3, L4, and L5 account for 10.2%, 14.7%, 15.4%, and 3.9%, respectively. This observed class imbalance should be considered when selecting training losses and evaluation measures. Metadata fields identify image ID, file path, label, camera, session, date, elapsed seconds, capture identifier, split, image dimensions, and checksum fields. Synthetic records add parent linkage and generator parameters. Representative real and synthetic images are shown in Fig. 2, Fig. 4, low-level characterization in Fig. 5, and the complete release workflow in Fig. 6.

## 4 Experimental Design, Materials and Methods

### 4.1 Study system and camera deployment

**Fig. 1:**
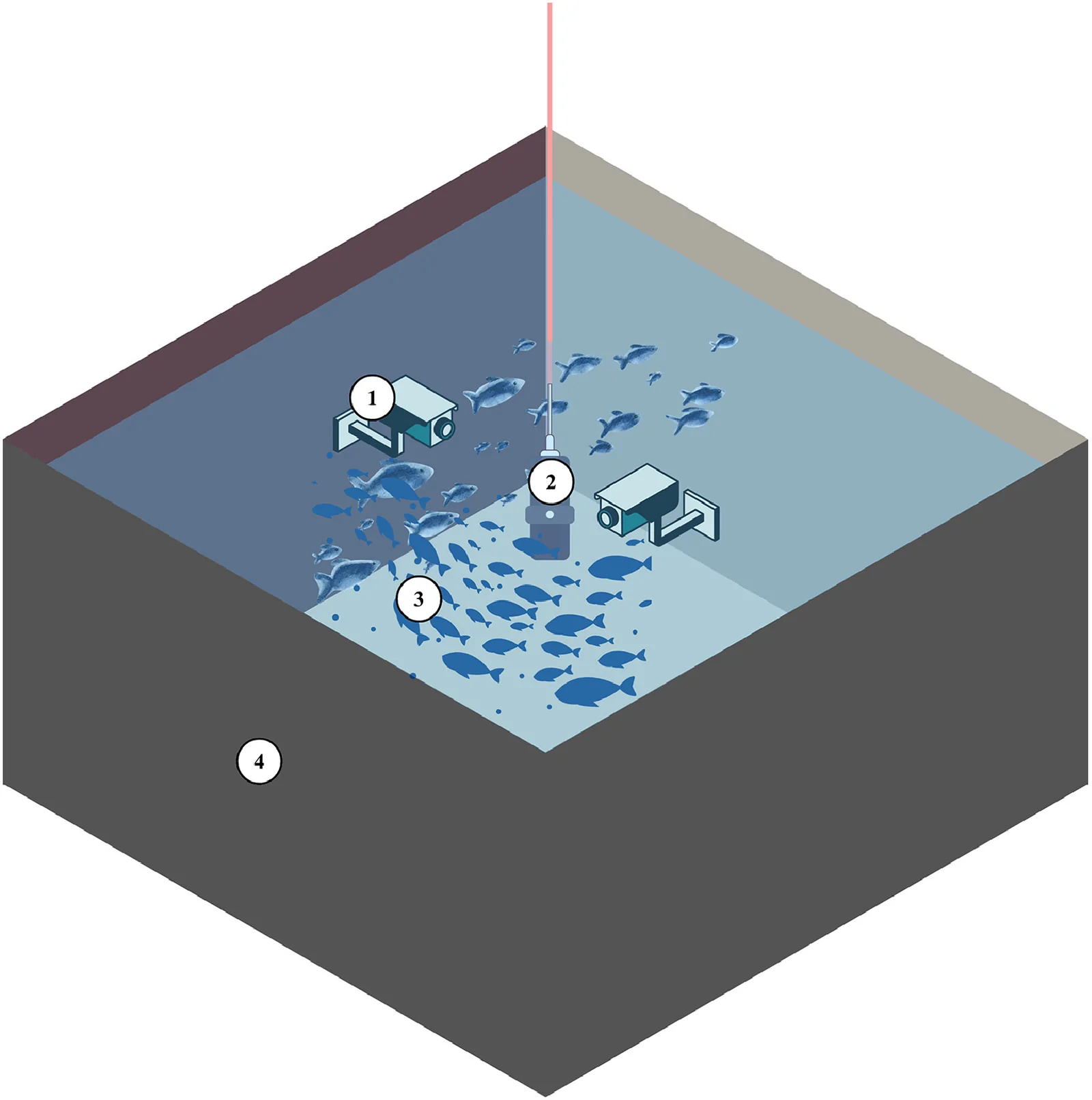
Data collection setup. Numbered elements indicate (1) the underwater camera node, (2) the suspended sensor/cable fixture, (3) the fish population, and (4) the tank environment.

Images were collected from 30 August to 19 September 2025 at the Research Institute for Aquaculture No. 1, Hai Phong, Viet Nam. The monitored system consisted of shallow cement rearing tanks used for golden pompano (*Trachinotus blochii*). Two fixed underwater camera nodes, CAMBOT1 and CAMBOT2, were deployed (Fig. 1). Each node used a Barlus IPC5MPIR-BX10 underwater camera mounted at approximately 1 m depth. The cameras were enclosed in IP68-rated 304 stainless-steel housings with tempered-glass viewports and used a 3.6 mm lens with an 85 degree horizontal field of view. Recording used ambient daylight only. The routine schedule included one morning session at 09:00 and one afternoon session at 16:00, each lasting approximately one hour. Source videos and extracted images had the same resolution, 3072 × 2048 pixels. After screening and curation, the dataset was derived from approximately 53 h of valid footage. Instrumental measurements of water quality, turbidity, temperature, and illumination were not collected with the videos.

### 4.2 Frame sampling and identifiers

Frames were sampled every 10 s from valid video footage. Each real image identifier encodes the camera, date, session, and elapsed second. A capture is defined as one camera-date-session unit. The real subset contains 18,972 images. The metadata fields include image identifier, origin, synthetic flag, label, source label, target label, camera, session state, date, elapsed second, capture identifier, group identifier, split, parent image identifier, dimensions, relative path, source filename, synthetic generator parameters, and checksum fields.

### 4.3 Capture-disjoint splits and leakage control

The official splits were assigned at the capture level rather than by individual frame. This design reduces leakage from neighbouring video frames and repeated scene content, which is a known issue in visual datasets with correlated scenes or spatiotemporal sampling [[17], [18], [19]]. The real-only split contains 14,932 training images from 50 captures, 1,883 validation images from six captures, and 2,157 test images from six captures. The real+synthetic training split adds 2,800 split-consistent synthetic images. The mixed validation and test files each add 400 split-consistent synthetic images. The primary evaluation domain is the real-only validation and test domain.

### 4.4 Label taxonomy

The taxonomy separates viewport fouling from water-column degradation. A frame with low contrast, suspended particles, or poor visibility can remain Level 1 if no stable surface deposit is visible. A frame can receive a higher level when stable material is attached to the viewport, even if the water column behind the surface is relatively clear.

### 4.5 Temporal annotation protocol

Single frames were labelled with temporal context because suspended material and attached deposits can look similar. Level 1 was anchored to the absence of visible stable deposits, while Level 5 was anchored to strong viewport obstruction. Documented mechanical cleaning reset the camera state. In the absence of visible cleaning evidence, viewport fouling was treated as non-decreasing over short time scales within a continuous capture. Three annotators independently labelled the images. Before adjudication, agreement was assessed using pairwise quadratic-weighted Cohen’s kappa across the five ordered levels; the recorded mean across the three annotator pairs was *κ_w_* = 0.87 [20]. Disagreements were reviewed using neighbouring frames, date-camera-session context, and visible cleaning events. The released label is the adjudicated consensus label.

### 4.6 Synthetic generation

Synthetic images were generated only for Levels 3-5 to supplement moderate-to-heavy fouling cases (Fig. 4). The subset contains 3,600 images: 540 Level 3, 900 Level 4, and 2,160 Level 5. Level 3 uses Level 2 parents before Level 1 fallback, Level 4 uses Level 3 before Level 2, and Level 5 uses Level 3, with Level 2 fallback restricted to training. Every synthetic image inherits its real parent’s official split. The 11 deposit assets are RGBA appearance-and-mask layers representing diatomaceous biofilm, aquatic bryophyte fouling, microphytobenthic diatoms, Ulva-like foliose macroalgae at several densities, sedimentation, exopolymeric film, particulate fouling, and benthic macroalgae. Four are author-prepared RGBA assets (A_0001-A_0003 and A_0010), and seven are procedural Blender assets (A_0004-A_0009 and A_0011). The assets are assigned to disjoint train, validation, and test pools.

For the procedural assets, generated texture coordinates were warped with a three-dimensional fBm Noise Texture (scale 18.0; mix factor 0.1). A two-dimensional Hybrid Multifractal Noise Texture and Cardinal-interpolated Color Ramp formed the alpha morphology (base scale 4.7; threshold 0.160), while a Linear-interpolated Color Ramp supplied green pigmentation. The Principled BSDF used roughness 1.0, transmission weight 0.719, and subsurface weight 0.215. Asset preparation is distinct from the subsequent image-overlay operation.

Let *A* and *M* denote an asset’s RGB layer and 8-bit alpha mask, with native dimensions *w_a_* × *h_a_*, and let the parent frame have dimensions *W* × *H*. The asset is scaled to cover 1.1 times the parent diagonal (with resized width capped at 4,000 pixels), rotated by *θ* sampled uniformly from the recorded integer range 0-360 degrees, centre-cropped to *W* × *H* using replicated borders, and downsampled then upsampled by integer factor *d* sampled from 15-35. With *S, R, C*, and *D* denoting these operations, the transformed RGB asset and mask are defined by Eq. (1).

(1) $$s_{0}=1.1\surd \left(W^{2}+H^{2}\right)/min\left(h_{a},w_{a}\right),s^{*}=min\left(s_{0},4000/w_{a}\right)$$

$$\left(\tilde{A},\tilde{M}\right)=D_{d}\left\{C_{W,H}\left[R_{\theta }\left(S_{s*}\left(A,M\right)\right)\right]\right\}$$

(2) $$\alpha \left(x,y\right)=o\tilde{M}\left(x,y\right)/255,o∼U\left(0.10,0.60\right)$$

(3) $$b=\left(1/WH\right)\Sigma_{x}\Sigma_{y}\alpha \left(x,y\right)$$

(4) $$I_{syn}\left(x,y,c\right)=clip\left\{I_{p}\left(x,y,c\right)+\alpha \left(x,y\right)\left[∼A\left(x,y,c\right)-I_{p}\left(x,y,c\right)\right],0,255\right\}$$

Here, *α* is the effective per-pixel opacity, *b* is mean viewport blockage, *I_p_* is the real parent image, and *c* indexes the RGB channels. The frozen blockage intervals *b* < 0.02, 0.02-0.08, 0.08-0.15, 0.15-0.25, and *b* ≥ 0.25 produce label increments 0, 1, 2, 3, and 4, respectively; the result is capped at Level 5. Outputs that do not reach the requested target label are rejected. The accepted image, parent, asset, *θ, s, d, o, b*, label increment, and random seed are recorded in metadata.

### 4.7 Low-level synthetic-image characterization

Fig. 5 uses all released real and synthetic images labelled L4 or L5. Each image is read as an 8-bit grayscale array *G* with *N* = WH pixels. Let *μ_G_* be mean brightness, *L* = Δ*G* the discrete Laplacian response, and *S_x_* and *S_y_* the 3 × 3 Sobel operators. Mean brightness and RMS contrast, Laplacian variance, Tenengrad, and 256-bin base-2 entropy are defined in Eqs. (5)-(8).

(5) $$\mu_{G}=\left(1/N\right)\Sigma_{i}G_{i},C_{RMS}=\surd \left[\left(1/N\right)\Sigma_{i}{\left(G_{i}-\mu_{G}\right)}^{2}\right]$$

(6) $$V_{L}=\left(1/N\right)\Sigma_{i}{\left(L_{i}-\mu_{L}\right)}^{2},L=\Delta G$$

(7) $$T=\left(1/N\right)\Sigma_{i}\left\{{\left[{\left(S_{x}*G\right)}_{i}\right]}^{2}+{\left[{\left(S_{y}*G\right)}_{i}\right]}^{2}\right\}$$

(8) $$H=-\Sigma_{j:p_{j}>0}p_{j}log_{2}\left(p_{j}\right)$$

(9) $$F_{n}\left(t\right)=\left(1/n\right)\Sigma_{i}1\left(x_{i}\leq t\right)$$

The PCA input comprises these four metrics plus *μ_G_*. Each feature is standardized over the combined real-severe and synthetic-severe set using its population mean and standard deviation before singular-value decomposition. For each displayed metric *x*, the empirical cumulative distribution is Eq. (9). The released reproduction outputs include every image-level statistic, feature-scaling value, PCA loading, and explained-variance value.

### 4.8 Code and software

**Fig. 2:**
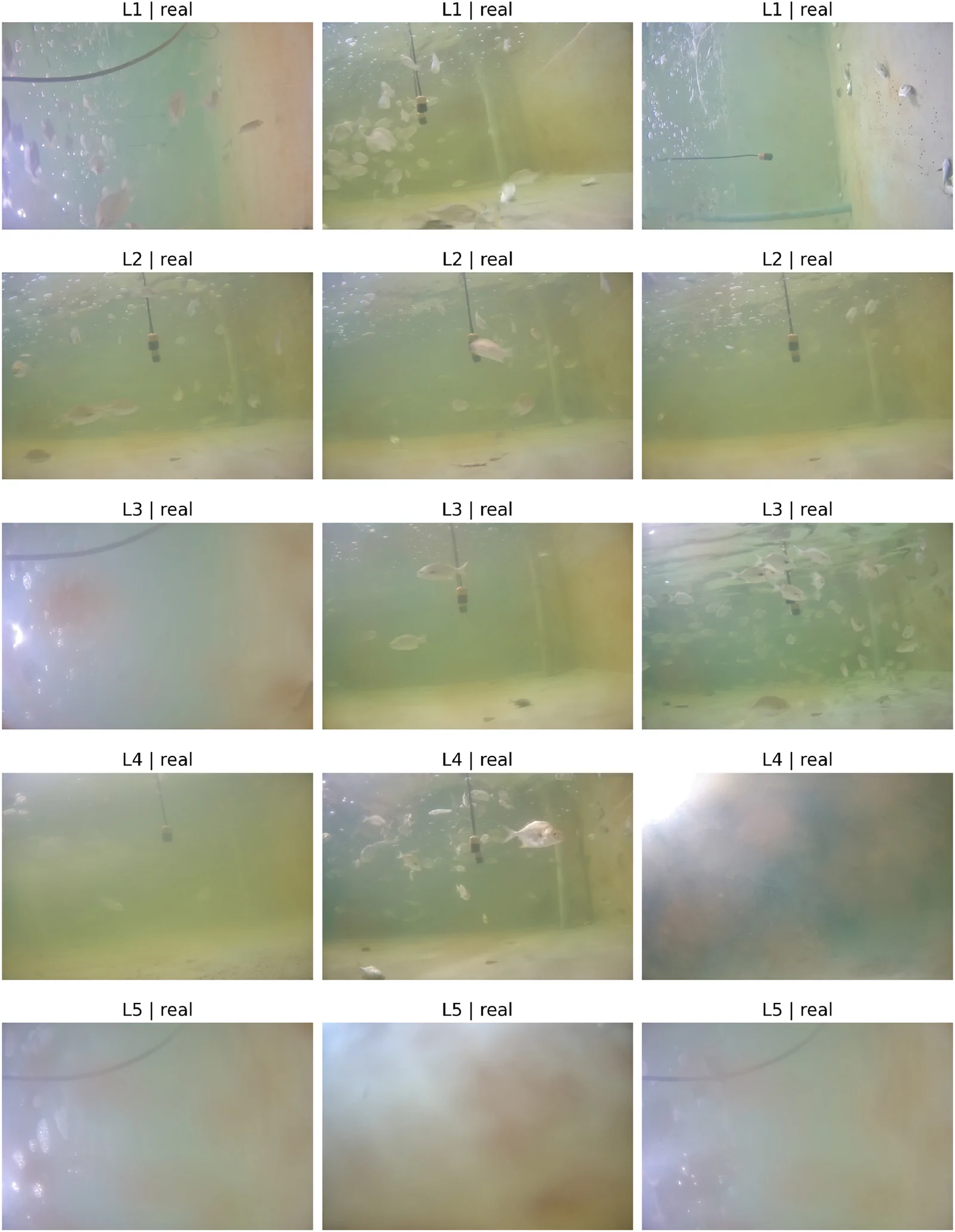
Representative real images grouped by the five viewport-fouling levels, L1-L5.

**Fig. 3:**
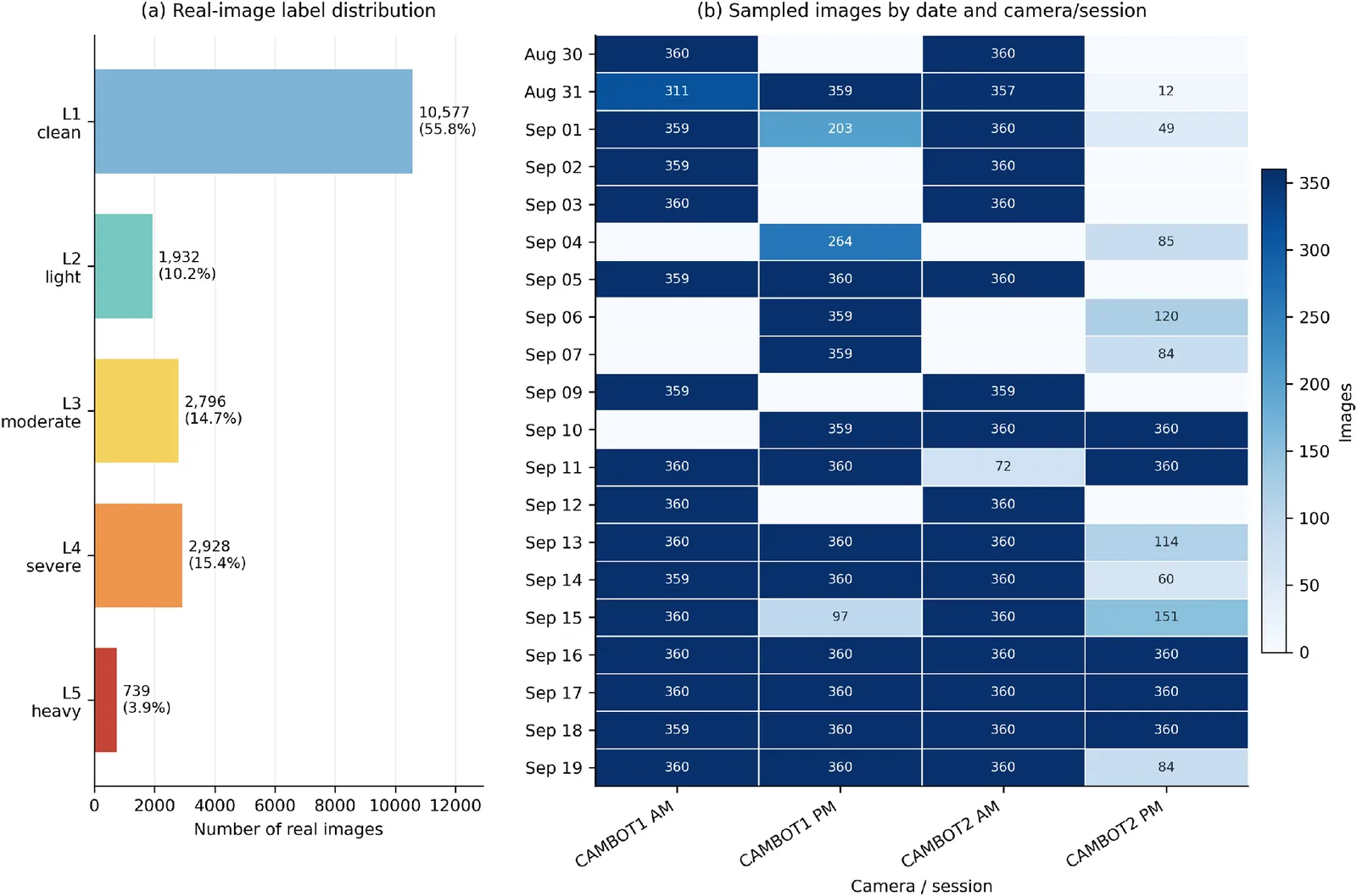
Composition of the real-image subset. (a) Real-image counts and percentages by label. (b) Number of sampled images by date and camera/session.

**Fig. 4:**
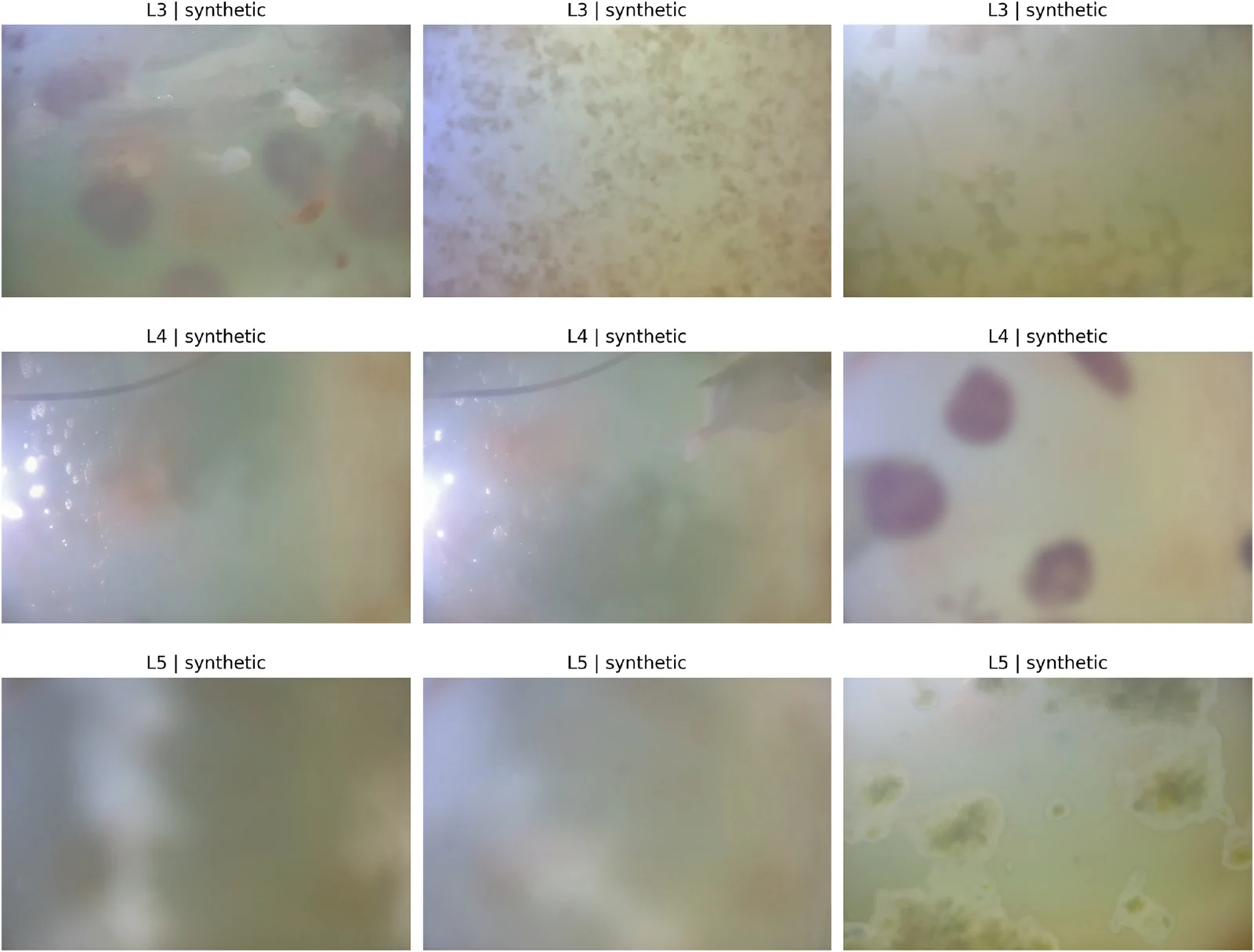
Examples of the synthetic viewport-fouling subset for Levels 3-5. Synthetic images preserve the parent scene and add controlled surface obstruction.

**Fig. 5:**
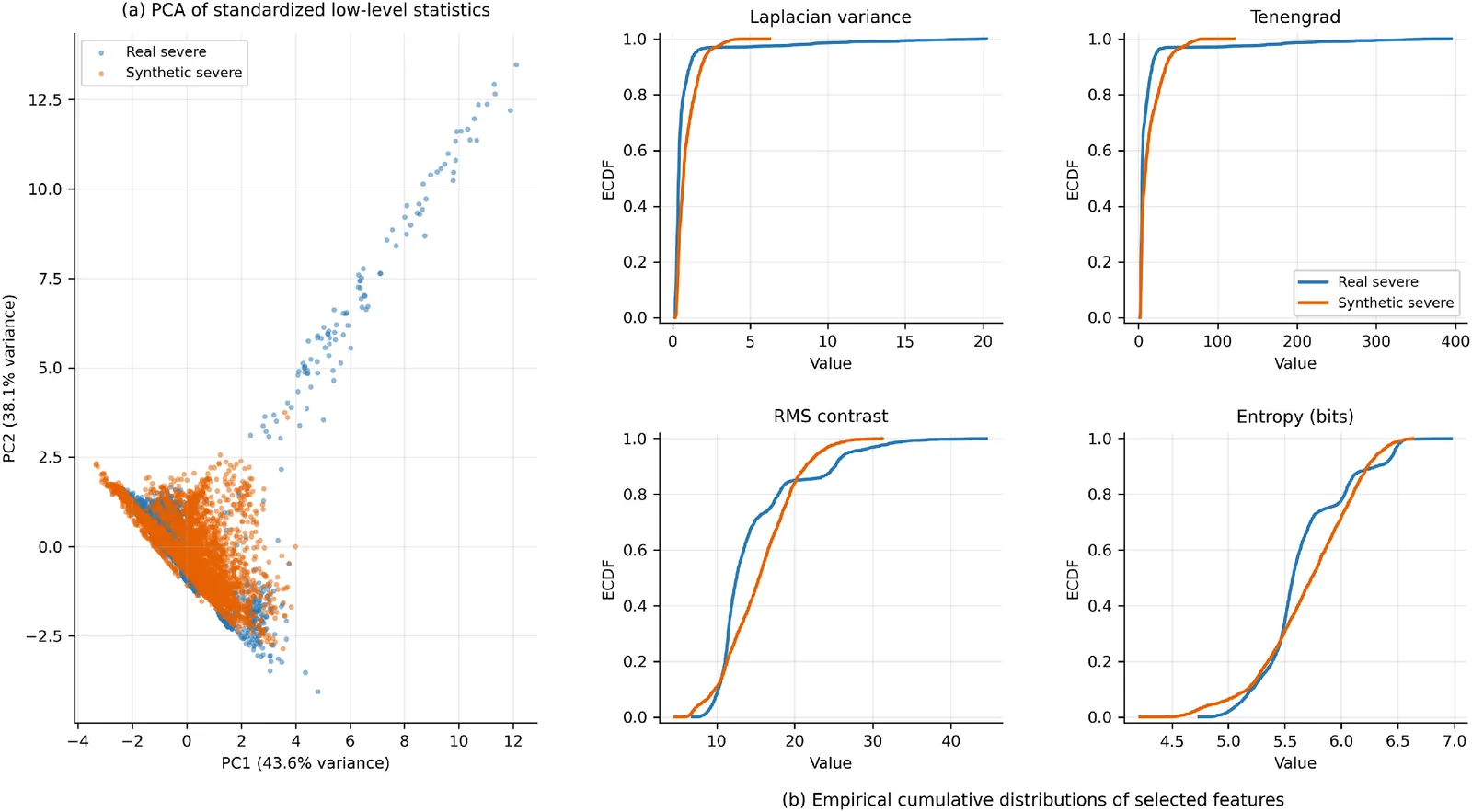
PCA and empirical cumulative distributions of low-level statistics for all released real severe (L4-L5; n = 3,667) and synthetic severe (L4-L5; n = 3,060) images. PCA uses standardized Laplacian variance, Tenengrad, RMS contrast, entropy, and mean brightness.

**Fig. 6:**
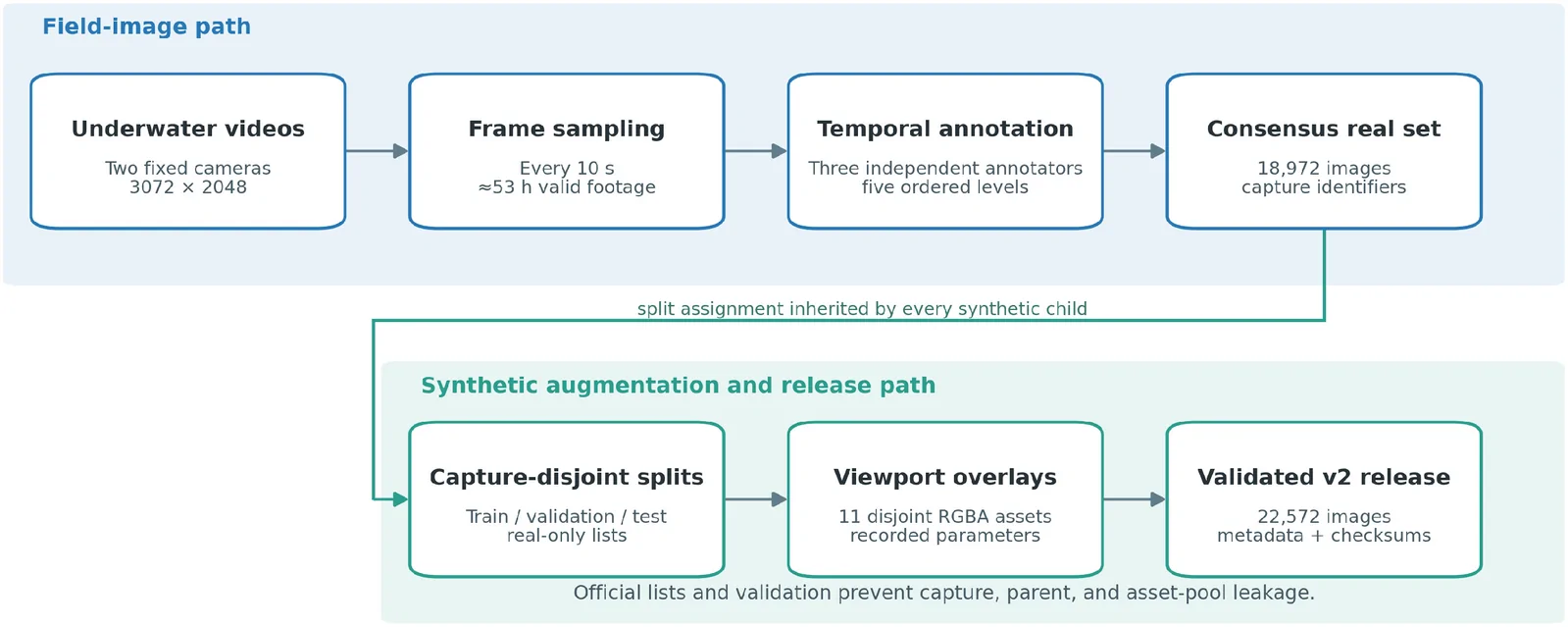
End-to-end CleanCam workflow from video acquisition and frame sampling through temporal annotation, capture-disjoint splitting, synthetic viewport overlays, and validated v2 release.

The public repository at https://github.com/khoa288/CleanCam [2] contains the analysis package, release builder and validator, a step-by-step quickstart, the data dictionary, fixed manuscript example identifiers, and scripts/reproduce_manuscript.py. One command regenerates Fig. 2, Fig. 3, Fig. 4, Fig. 5, Fig. 6 and Table 1, Table 2, Table 3 and saves the source statistics, PCA loadings, explained variance, software versions, and output checksums. The frozen core environment uses Python 3.11, NumPy 1.26.4, pandas 2.2.3, OpenCV 4.10.0.84, Pillow 10.4.0, Matplotlib 3.9.2, scikit-learn 1.5.2, SciPy 1.14.1, and tqdm 4.66.5. PyTorch benchmark dependencies are provided separately. An optional training entry point uses the official capture-disjoint splits to fine-tune MobileNetV2, ResNet-18, or EfficientNet-B0 with either a standard five-class classification head trained using cross-entropy or CORAL/CORN ordinal heads, and exports checkpoints, predictions, and evaluation summaries.

**Table 1:** Folder and file structure of the CleanCam release.

Path	Contents and purpose
images/real/	Curated field-captured RGB JPEG images, organized by ordinal severity label.
images/synthetic/	Generated RGB JPEG images for Levels 3-5, linked to real parents and inherited splits.
assets/dirt_assets/	Eleven RGBA deposit assets assigned to disjoint train, validation, and test pools.
code/	Dataset-release builder and release-validation scripts.
metadata/metadata.csv	Master image-level metadata table for real and synthetic images.
metadata/metadata_real.csv	Metadata records for real images only.
metadata/metadata_synthetic.csv	Synthetic records including parent linkage and generator parameters.
metadata/dirt_assets_manifest.csv	Asset descriptions, preparation categories, dimensions, split pools, and checksums.
documentation/	Quickstart, data dictionary, label taxonomy, acquisition conditions, and deposit-asset method.
metadata/split_summary.csv	Counts for each official real-only and real-plus-synthetic split.
metadata/build_summary.json; metadata/file_manifest_sha256.csv	Release settings, validation summary, and complete file checksums.
splits/official/	Official train, validation, and test CSV files for real-only and real-plus-synthetic variants.

**Table 2:** Dataset composition by origin and ordinal severity level.

Subset	Total	L1	L2	L3	L4	L5
Real	18,972	10,577	1,932	2,796	2,928	739
Synthetic	3,600	0	0	540	900	2,160
Combined	22,572	10,577	1,932	3,336	3,828	2,899

**Table 3:** Five-level ordinal viewport-fouling label taxonomy.

Level	Viewport condition	Cleaning interpretation
L1 clean	Clean viewport. Visibility may still be affected by water, particles, or lighting.	No cleaning is indicated by the image alone.
L2 light	Light local deposits, with the view still largely usable.	Cleaning is not urgent; continue monitoring.
L3 moderate	Clear deposits affecting part of the field of view.	Inspect or clean if the condition persists or conflicts with the monitoring task.
L4 severe	Severe deposits or smearing that interfere with routine interpretation.	Cleaning is recommended before routine image-based monitoring continues.
L5 heavy	Heavy obstruction or blur that strongly compromises scene interpretation.	Cleaning is required before treating the image stream as reliable.

## Limitations

CleanCam should be reused with attention to scope. All real images were collected at one aquaculture facility, from two fixed cameras, one tank type, one deployment period, and one farmed species. Users should validate trained models across facilities, seasons, camera housings, water types, lighting conditions, cleaning practices, and fouling communities before operational deployment. The labels separate viewport-attached fouling from water-column degradation using visual and temporal evidence, but the release does not include direct measurements of turbidity, suspended solids, chlorophyll, illumination, water chemistry, or deposit composition. The synthetic subset is provided for controlled augmentation and stress testing, not as field evidence or as a calibrated model of biofouling growth. The dataset provides image-level ordinal labels rather than segmentation masks, deposit taxonomy, cleaning-cost records, or direct maintenance outcomes. Adjacent-level ambiguity remains possible, especially near the Level 3/Level 4 boundary.

## Credit Author Statement

**Khoa Nguyen:** Conceptualization, Methodology, Software, Formal analysis, Validation, Visualization, Writing - original draft, Writing - review and editing. **Anh Hoang:** Conceptualization, Methodology, Data curation, Software, Validation, Visualization, Writing - original draft, Writing - review and editing. **Nhat-Anh Tran:** Data curation, Software, Investigation, Validation, Writing - review and editing. **Nguyet-Anh Tran:** Data curation, Investigation, Validation, Writing - review and editing. **Minh-Hoang Pham:** Investigation, Resources, Data curation. **Khoi Phan:** Investigation, Resources, Data curation. **Van-Dinh Nguyen:** Project administration, Funding acquisition. **Dzung V. Dinh:** Project administration, Funding acquisition. **Cuong Do:** Conceptualization, Supervision, Project administration, Funding acquisition, Writing - review and editing.

## Ethics Statement

The authors have read and followed the ethical requirements for publication in Data in Brief. This work did not involve human subjects or data collected from social media platforms. No experimental procedures were performed on animals for the purpose of this dataset. The data were collected through non-invasive passive underwater imaging during routine aquaculture monitoring of golden pompano (*Trachinotus blochii*). The imaging did not involve physical handling, experimental manipulation, changes to diet, changes to routine husbandry, or animal-level outcome measurements. Site permission was obtained for camera deployment and public release of the dataset. The sex of the fish was not recorded because the dataset contains image-level camera-condition labels and does not analyze animal-level or sex-dependent outcomes.

## Ethics Declaration

Ethics committee approval was not required. The dataset was collected through non-invasive passive underwater imaging during routine aquaculture monitoring. No animals were handled or experimentally manipulated, and no changes were made to diet, husbandry, or normal farm operations. The study assessed camera-viewport condition rather than animal-level outcomes. Site permission was obtained for camera deployment and public release of the data.

## Data Availability

ZenodoCleanCam: a labelled image dataset for camera-cleaning decisions in aquaculture monitoring (Original data).
